# miR-506 functions as a tumor suppressor in glioma by targeting STAT3

**DOI:** 10.3892/or.2021.8212

**Published:** 2021-10-29

**Authors:** Tao Peng, Lixiang Zhou, Ling Zuo, Yongxin Luan

Oncol Rep 35: 1057-1064, 2016; DOI: 10.3892/or.2015.4406

Following the publication of this paper, it was drawn to the Editors attention by a concerned reader that certain of the western blot assay data shown in Figs. 4D, 6B and 7F were strikingly similar to data appearing in different form in other articles by different authors. Furthermore, an independent investigation of this paper conducted by the Editorial Office unveiled possible anomalies associated with the cyclin D1 data presented in Fig. 4D. Owing to the fact that the contentious data in the above article were already under consideration for publication, or had already been published, prior to its submission to *Oncology Reports*, the Editor has decided that this paper should be retracted from the Journal. The authors themselves requested that the paper be retracted. The Editor apologizes to the readership for any inconvenience caused.

